# The dynamic relationship between inorganic polyphosphate and adenosine triphosphate in human non‐small cell lung cancer H1299 cells

**DOI:** 10.1002/2211-5463.13753

**Published:** 2024-01-01

**Authors:** Kaori Tsutsumi, Thititip Tippayamontri, Mari Hayashi, Nobuto Matsuda, Yusaku Goto

**Affiliations:** ^1^ Department of Biomedical Science and Engineering Faculty of Health Sciences Hokkaido University Sapporo Japan; ^2^ Department of Radiological Technology and Medical Physics Faculty of Allied Health Sciences Chulalongkorn University Bangkok Thailand; ^3^ Department of Health Sciences, School of Medicine Hokkaido University Sapporo Japan

**Keywords:** adenosine triphosphate, human non‐small cell lung cancer cells, inorganic polyphosphate, mitochondria, mitochondrial membrane potential

## Abstract

Inorganic polyphosphate (polyP) plays a vital role in cellular energy metabolism and signaling, owing to its structure and high‐energy phosphate bonds. Intracellular ATP functions both as a cellular energy source and a key factor in cell death, and ATP dynamics in tumor cells are crucial for advancing cancer therapy. In this study, we explored the interplay between polyP and ATP in cellular energy metabolism. Treatment with polyP did not affect cell proliferation of human non‐small cell lung cancer H1299 and human glioblastoma T98G cell lines as compared to their respective control cells until 72 h post‐treatment. The mitochondrial membrane potential (MMP) in polyP‐treated cells was low, contrasting with the time‐dependent increase observed in control cells. While the ATP content increased over time in untreated and Na‐phosphate‐treated control cells, it remained unchanged in polyP‐treated cells. Furthermore, the addition of cyclosporine A, a mitochondrial permeability transition pore (mPTP) inhibitor, failed to restore ATP levels in polyP‐treated cells. We performed lactate assays and western blot analysis to evaluate the effect of polyP on glucose metabolism and found no significant differences in lactate secretion or glucose‐6‐phosphate dehydrogenase (G6PD) activity between polyP‐treated and control cells. Additional pyruvate restored MMP but had no effect on the cellular ATP content in polyP‐treated cells. We observed no correlation between the Warburg effect and glucose metabolism during ATP depletion in polyP‐treated cells. Further investigation is warranted to explore the roles of polyP and ATP in cancer cell energy metabolism, which might offer potential avenues for therapeutic interventions.

## Graphical abstract

Exogenous polyP‐treated tumor cells showed ATP depletion concomitant with a decrease in MMP. Additional pyruvate restored MMP but had no effect on the cellular ATP content. It did not affect the Warburg effect or glucose metabolism. PolyP may have therapeutic potential for tumor through its association with ATP metabolism, OXPHOS, Ca^2+^, MMP, and cellular energy metabolism.

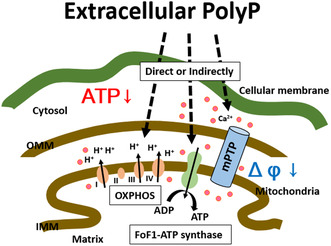

**Abbreviations**
ATP: adenosine triphosphate
CsA: Cyclosporine A
ERK: extracellular‐signal‐regulated kinase
FGF‐2: basic fibroblast growth factor
G6PD: glucose‐6‐phosphate dehydrogenase
MAPK: mitogen‐activated protein kinase
MMP: mitochondrial membrane potential
mPTP: mitochondrial permeability transition pore
mTOR: mammalian target of rapamycin
OXPHOS: oxidative phosphorylation
PHB: polyhydroxybutyrate
polyP: inorganic polyphosphate
PPP: pentose phosphate pathway

Inorganic polyphosphate (polyP) is a linear polymer comprising tens to hundreds of orthophosphate residues linked by high‐energy phosphoanhydride bonds [1, 2]. It exists in all living organisms, ranging from bacteria to mammals, and plays a vital role in various cellular processes, including cell signaling, gene regulation, stress responses, and enzyme regulation [1, 2]. It also serves as a potential reservoir of phosphate groups utilized for both energy storage and regulatory purposes [1, 2, 3, 4, 5]. In addition, recent studies have shown numerous physiological and remarkable functions of polyP as a potential drug [6, 7, 8, 9, 10, 11]. In our previous study, we reported the polyP treatment in non‐small lung cancer cell lines, specifically H1299, increased the number of double‐stranded breaks after X‐ray irradiation and enhanced their radiosensitivity [12].

PolyP and adenosine triphosphate (ATP) are considered crucial molecules in cellular energy metabolism and signaling. Owing to its chemical structure with high‐energy phosphate bonds, polyP is believed to play crucial interconnected roles in various cellular processes, primarily through its involvement in energy transfer and utilization, often in conjunction with ATP. A previous study reported that the ATP content was relatively low in polyp‐treated cells, thus indicating an interplay between polyP and ATP in cellular metabolism [12]. Meanwhile, polyP plays a role in the activation of mitochondrial permeability transition pore (mPTP) via association with ATPase C‐subunit and polyhydroxybutyrate (PHB) [13, 14]. First, Seidlmayer *et al*. demonstrated that the interaction between polyP and Ca^2+^ mediates mPTP opening, a process of physiological significance in cellular stress and cardiac cell death [15, 16]. Elustondo *et al*. [14] proposed that calcium‐induced mPTP opening is associated with the formation of a complex involving the ATPase C‐subunit, PHB, and polyP. Subsequently, Solesio *et al*. hypothesized that polyp serves as a structural component of the mPTP pore, emphasizing its potential role in modulating mPTP activity through calcium activation, selectivity, and voltage‐dependence [13]. Furthermore, Seidlmayer *et al*. [17] highlighted the dual role of polyP as an energy metabolism resource and an mPTP activator depending on its length. ATP is primarily generated through mitochondria oxidative phosphorylation (OXPHOS) processes within the mitochondria [18]. However, the opening of mPTP by the leak currents in inner mitochondrial membrane disrupts the coupling of ATP synthase activity [19]. The mitochondrial function, including mPTP opening and ATP synthesis, is closely associated with ATP metabolism [19]. PolyP can modulate ATP metabolism by regulating both the formation and opening of mPTP, which are crucial for sustaining mitochondrial OXPHOS. In fact, Hambardikar *et al*. [9] suggested that the absence of polyP in mitochondria leads to increased reactive oxygen species (ROS) levels due to OXPHOS dysregulation.

The relationship between polyP and ATP levels is multifaceted. As discussed above, PolyP can influence ATP synthesis and utilization by regulating key metabolic pathways and enzyme activities crucial for energy production. Moreover, Pavlov *et al*. and Baev *et al*. have separately proposed a hypothesis that polyP is produced by mitochondrial Fo‐F1 ATP synthase [20, 21, 22], an enzyme that catalyzes ATP synthesis using the electrochemical proton gradient across the mitochondrial inner membrane while also generating a proton gradient to produce energy [22]. This enzyme plays a crucial role in maintaining the mitochondrial membrane potential and facilitating ATP production within the mitochondria [22]. PolyP potentially modulates various processes, such as glycolysis, OXPHOS, and other metabolic pathways, by regulating ATP levels in conjunction with the activity of Fo‐F1 ATP synthase and the opening of mPTP [22]. Boyineni *et al*. [11] reported the properties of polyP as an energy source for tumorigenesis in mouse brain tumor‐initiating cells. Interestingly, in such cell, under the conditions of ATP depletion due to the suppression of glycolysis and OXPHOS during glucose starvation, polyP is utilized as an alternative energy source instead of ATP, leading to a decrease in polyP accumulation [11]. However, the effect of extracellular polyP as a drug, when added to cell and tissue culture media, may differ from that of endogenous polyP. Müller *et al*. [23] reported an increase in ADP and ATP levels induced by extracellular polyP in human osteogenic sarcoma cells. This finding contrasts with the results of Solesio *et al*. [8], who demonstrated that the depletion of endogenous polyP in human embryonic kidney 293 (HEK293) cells leads to ATP depletion. Our previous study also suggested that polyP treatment does not lead to an increase in the cellular ATP content in the mouse osteoblast‐like cell line MC3T3‐E1, and the human non‐small lung cancer cell line H1299 [12].

PolyP has been implicated in the regulation of ATP‐dependent processes, such as ion channels, membrane transporters, and protein phosphorylation [21, 24, 25]. It also interacts with the mammalian target of rapamycin (mTOR) [26] and influences mitochondrial metabolism [27]. The density of mitochondria increases when mitochondrial polyP is depleted by expressing the yeast‐derived exopolyphosphatase in mitochondria [28]. Our previous reports also suggested that polyP‐treated mouse osteoblastic cells exhibit low mitochondrial density in electron microscopy [29, 30]. They collectively suggest that polyP plays a significant role both as an extracellular drug and in endogenous physiological processes. The subtle balance of the polyP content in the cytosol, mitochondria, and outer membrane may indeed be associated with ATP metabolism. Solesio *et al*. [8] have emphasized the significance of endogenous mitochondrial polyP, which can induce a metabolic shift from oxidative phosphorylation to glycolysis. In addition, polyP plays a role in various cellular processes, including oxidative stress response, cell metastasis and neovascularization, cell apoptosis, and cell hemostasis, showcasing potential as a promising therapeutic agent [31, 32, 33].

In this study, we investigated the dynamic relationship between polyP and ATP, highlighting their roles in cellular energy metabolism. In addition, we explored the roles of polyP and ATP in cancer, which may offer potential avenues for therapeutic interventions.

## Results and Discussion

### Effect of polyP on tumor cell growth

**Fig. 1:**
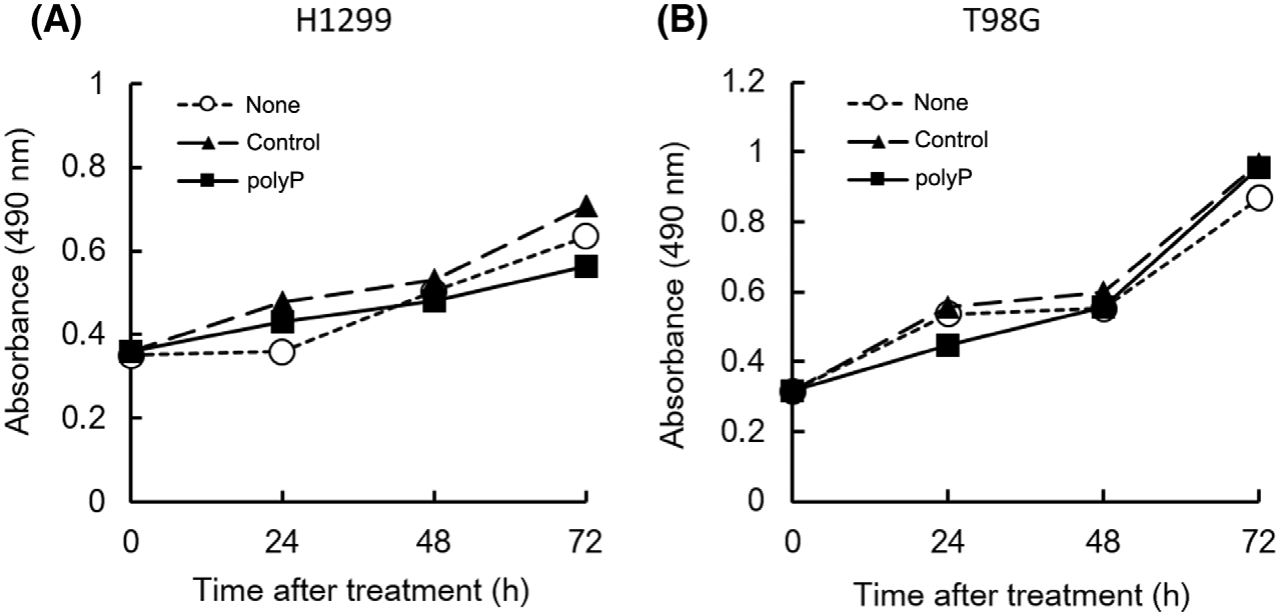
Cell growth. The cell growth of H1299 (A) and T98G (B) cells was monitored using an MTS assay. The white circle (○) represents the untreated case (none), the black triangle (▲) represents 1 mm sodium phosphate (pH 6.8) as a control for polyP, and the black square (■) represents 1 mm polyP. The plots represent the mean (± SD) of sextuplicate experiments.

PolyP has emerged as a potential regulator of cancer cell death and plays a significant role in modulating cell fate [12, 34, 35]. Initially, we performed an MTS assay to assess cellular proliferation in polyP‐treated cells compared to the control groups (“none” and “control”). In this study, we utilized two different control groups for polyP treatment: “none,” which contained the same volume of MilliQ as the additive polyP, and “control,” which contained the same volume of sodium phosphate buffer (pH 6.9) as the additive polyP. Furthermore, for comparison with our previous study, we selected two different tumor cell lines, the human lung cancer cell line H1299 and the human glioblastoma cell line T98G, both lacking the *p53* gene. Notably, treatment with polyP did not affect the growth of H1299 cells until 72 h post‐treatment (Fig. 1A). This phenomenon was consistent with the T98G cell line used in this study (Fig. 1B), and it also aligned with the findings of our previous study involving the same cell line [29]. In fibroblast‐derived cell lines, polyP promotes cell proliferation by modulating the activity of basic fibroblast growth factor (FGF‐2) [25]. Because FGF‐2 enhances cellular proliferation, migration, differentiation, and angiogenesis [36], polyP facilitates the binding of FGF‐2 to its receptors promotes cell growth activity [25]. However, because FGF‐2 expression has already been evaluated in advanced non‐small cell lung cancer and human glioma [37, 38, 39], polyP may not significantly affect the tumor cell growth (Fig. 1A,B). However, the rate of cellular FGF‐2 production during polyP treatment varies depending on multiple factors, including cell type, polyP concentration, treatment duration, and experimental conditions. The observed reduction in cell growth at 72 h after polyP treatment suggests that polyP may alter cellular responses and pathways involved in protein production, secretion, and signaling, potentially affecting FGF‐2 production in cancer cells. Furthermore, we explored the potential effects of pyruvate supplementation on cell metabolism, growth, and protein production. Pyruvate can provide additional carbon sources for cellular processes and influence the availability of energy substrates. In some cases, pyruvate supplementation may lead to enhanced cell growth by upregulating protein production and secretion, including the production of growth factors, such as FGF‐2.

PolyP has been shown to activate the ERK/MAPK pathway in different cell types [31, 40], which subsequently triggers downstream signaling events involving cellular proliferation, differentiation, and survival. Additionally, PolyP not only inhibits FGF‐2‐induced proliferation and extracellular‐signal‐regulated kinase (ERK)/p38 mitogen‐activated protein kinase (MAPK) activation in human endothelial cells but also blocks the binding of FGF‐2 to its cognate cell‐surface receptor [31]. Interestingly, pyruvate supplementation has been found to enhance ERK/MAPK activation and promote survival in cardiac myocytes [40]. Therefore, the combination of polyP and pyruvate in the ERK/MAPK pathway could have potential implications for various physiological and pathological conditions related to cancer development.

### ATP content in polyP‐treated cells

**Fig. 2:**
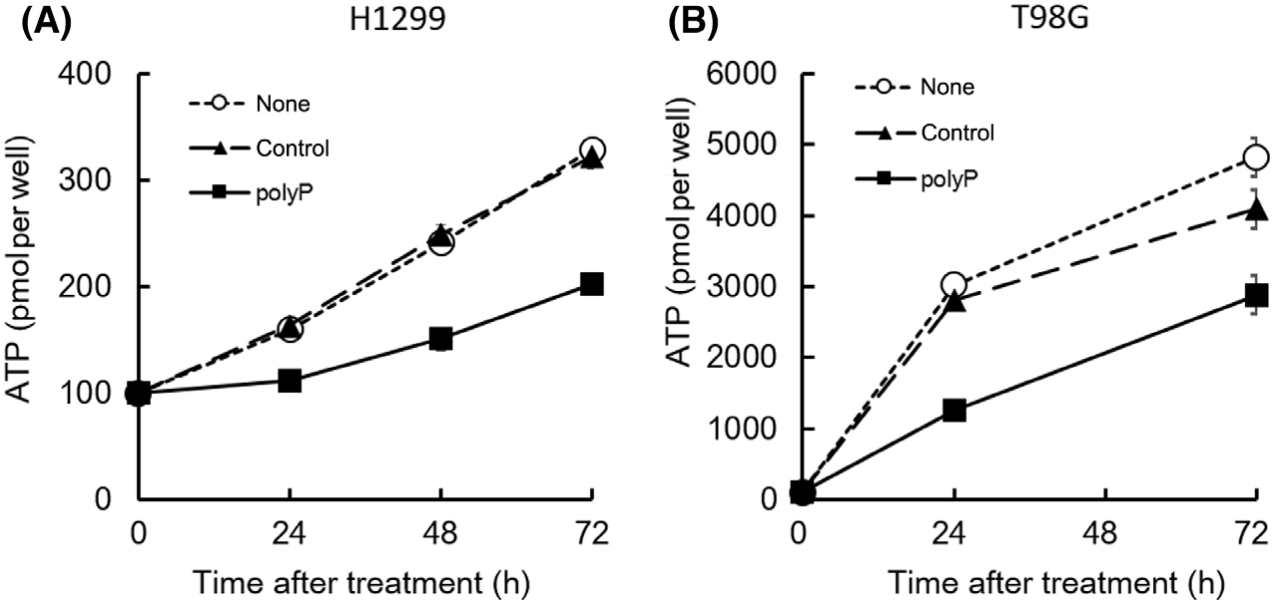
The change in cellular adenosine triphosphate content. ATP levels were measured using the CellTiter‐Glo® 2.0 Assay kit (Promega Corporation) in H1299 (A) and T98G (B). The white circle (○) represents the untreated case (none), the black triangle (▲) represents 1 mm sodium phosphate (pH 6.8) as a control for polyP, and the black square (■) represents 1 mm polyP. The plots represent the mean (± SD) of sextuplicate experiments.

PolyP influences ATP levels in tumor cells by interacting with cellular processes related to energy metabolism, specifically by modulating the activity of ATP synthase, the enzyme responsible for ATP production in the mitochondria [21, 22]. Previous studies have shown that polyP can directly interact with ATP synthase, inhibiting its activity and subsequently reducing ATP production [20, 41]. In our previous study, we reported that the cellular ATP content did not increase after X‐ray irradiation in polyP‐treated cells, which led to the upregulation of their radiosensitivity [12]. Figure 2 shows that ATP content increased in both untreated and sodium phosphate‐treated control cells over time. However, in polyP‐treated cells, this increase occurred independently of the cell type and without X‐ray irradiation (Fig. 2A,B). The variations in ATP levels among different cell types can be attributed to the varying amounts of endogenous ATP present in each cell type. ATP depletion in polyP‐treated cells has also been reported in a previous study involving the mouse osteoblastic cell line MC3T3‐E1 [29]. Our findings indicate that while pyruvate serves as a substrate in cellular energy metabolism and can contribute to ATP production, there are situations where pyruvate may not effectively restore ATP levels in cancer cells. This suggests that polyP may have the potential to inhibit the increase in intracellular ATP levels.

### Effect of polyP on mitochondrial membrane potential in H1299 cells

**Fig. 3:**
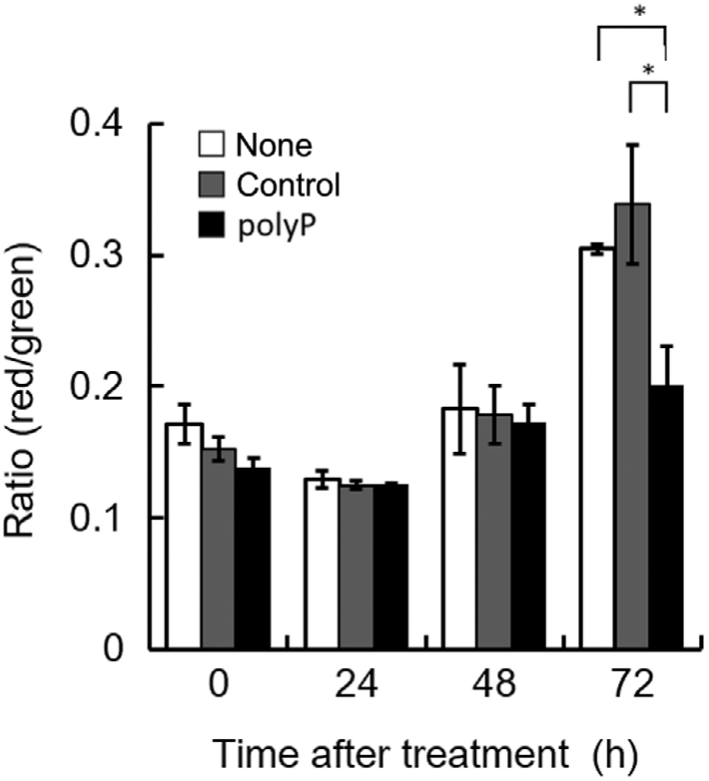
Change in mitochondrial membrane potential. Mitochondrial membrane potential was compared among three groups: none, control, and polyP. The white bar represents the untreated case (none), the gray bar represents 1 mm sodium phosphate (pH 6.8) as a control for polyP, and the black bar represents 1 mm polyP. Values represent the mean (± SD) of sextuplicate experiments. *P < 0.05 versus controls. The *P*‐values were determined by one‐way analysis of variance (ANOVA) and Tukey's honestly significant difference (HSD) test.

Recent insights suggest that polyP affects the reduction in mitochondrial membrane potential (MMP) by regulating the opening of the mPTP, in conjunction with the C‐subunit of ATPase and polyhydroxybutyrate (PHB) [14]. The effect of polyP on the mPTP in H1299 cells is an area of interest for understanding the molecular mechanisms underlying cellular responses to polyP treatment, especially concerning mitochondrial function and cell death pathways. Therefore, we subsequently performed an MMP assay. Up to 48 h after treatment with polyP, no significant differences in MMP were observed among the three groups; however, at 72 h, the MMP increased in the control groups, whereas the polyP‐treated cells showed no such increase in MMP (Fig. 3). These findings indicate that polyP influences the cellular MMP potential and leads to ATP depletion in tumor cells. Considering polyP's ability to regulate MMP associated with mPTP opening, in conjunction with the polyP‐PHB‐C‐subunit of the ATPase complex [13, 14], these results suggest that ATP depletion in polyP‐treated cells might result from a decrease in MMP, affected by mPTP opening associated with the polyP‐PHB‐C‐subunit of the ATPase complex.

### Effect of cyclosporine a (CsA) on ATP depletion

**Fig. 4:**
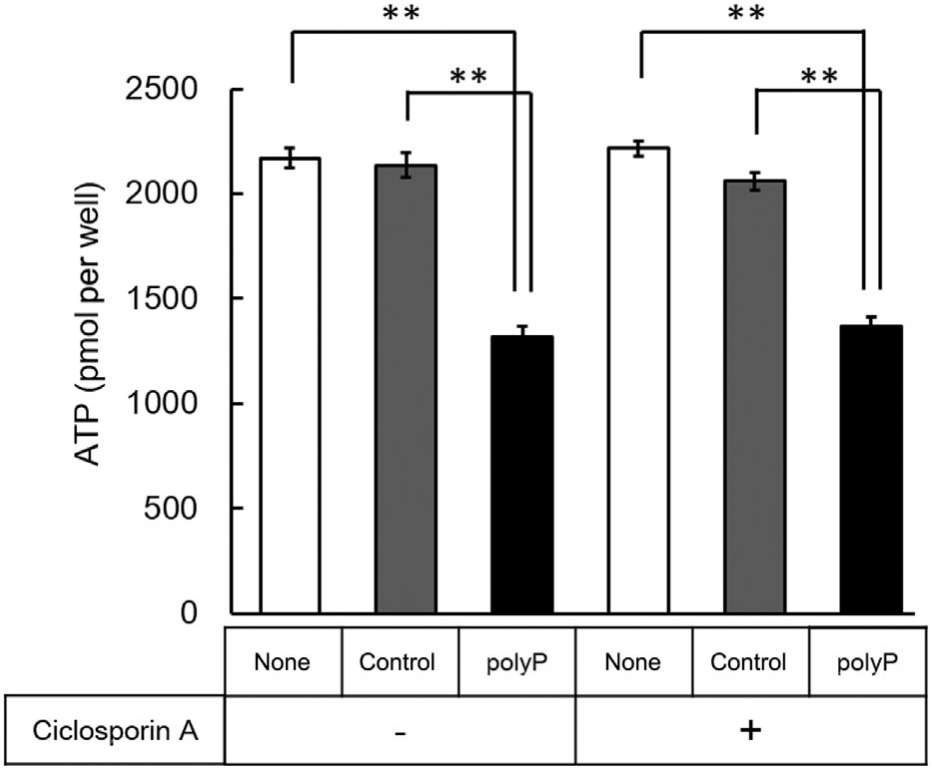
Cellular ATP content with or without 200 nm of cycloporine A (CsA) in H1299 cells. The cellular ATP content was measured 72 h after polyP treatment. In the assay group without CsA, an equal volume of ethanol was added to account for the CsA solvent. Values represent the mean (± SD) of sextuplicate experiments. **P < 0.01 versus controls. The *P*‐values were determined by one‐way analysis of variance (ANOVA) and Tukey's honestly significant difference (HSD) test.

Cyclosporine A (CsA), a well‐known mPTP inhibitor, suppresses mPTP opening by inhibiting the regulation of cyclophilin D (Cyp D) and calcium homeostasis [42, 43]. Owing to this property, CsA also significantly inhibits ATP depletion resulting from mPTP opening. Therefore, we conducted an ATP assay with the addition of CsA, expecting the restoration of cellular ATP levels through mPTP inhibition. Surprisingly, despite CsA's role as an mPTP inhibitor, its addition did not lead to the recovery of ATP depletion in polyP‐treated cells (Fig. 4). Further measurement conducted at 24 and 48 h after CsA treatment did not reveal any observed recovery in cellular ATP levels (data not shown). These results indicated that ATP depletion in polyP‐treated cells may not be attributed to causes other than mPTP opening by polyP. Solesio *et al*. [8] reported that a decrease in polyP by exopolyphosphatase in the mitochondria led to a decrease in ATP content. These results may appear to be in direct contrast to ours, which showed that polyP‐treated cells inhibited the increase in cellular ATP. However, their experiment differed in that they degraded endogenous polyP in mitochondria using PPX, whereas in our present study, we introduced exogenous polyP to the culture medium. There is a possibility that the reduction in MMP resulting from polyP treatment in our study might have occurred through a pathway distinct from the regulation of mPTP activity via interactions with F1‐ATPase and PHB in the inner mitochondrial membrane. For instance, this might involve a redirection of ATP metabolism away from OXPHOS or indirect changes in cellular Ca^2+^ levels induced by the addition of polyP. However, considering the potential of polyP to regulate ATP metabolism depending on the intricate intracellular environment, our current findings, juxtaposed with those of Solesio *et al*., might represent different facets of the same phenomenon, specifically the effect of polyP on mPTP activity and cellular ATP levels. The state of mPTP opening and the activity of F_0_F_1_‐ATPase may fluctuate between open/close or synthesis/hydrolysis states, depending on the balance of the cellular state and the Ca^2+^concentration. Thus, polyP might contribute to the regulation of delicate homeostasis within an organism [14, 15, 16, 19, 21, 22]. Recently, it has been reported that F_0_F_1_‐ATPase also plays a role in polyP synthesis and hydrolysis [21], and it is possible that intracellular ATP is exquisitely controlled by the balance between the amount of polyphosphate inside and outside of the mitochondria. Furthermore, considering Solesio *et al*.'s use of the human embryonic kidney cell line HEK293, which retains the functional *p53* gene, we must acknowledge the possibility that variations in *p53* status could contribute to differing effects of polyP on ATP levels. The p53 protein is a well‐known tumor suppressor that reacts to various cellular stressors. Recent studies have further highlighted its role in regulating energy metabolism and oxidative stress [44].

### Effect on the glucose metabolism

**Fig. 5:**
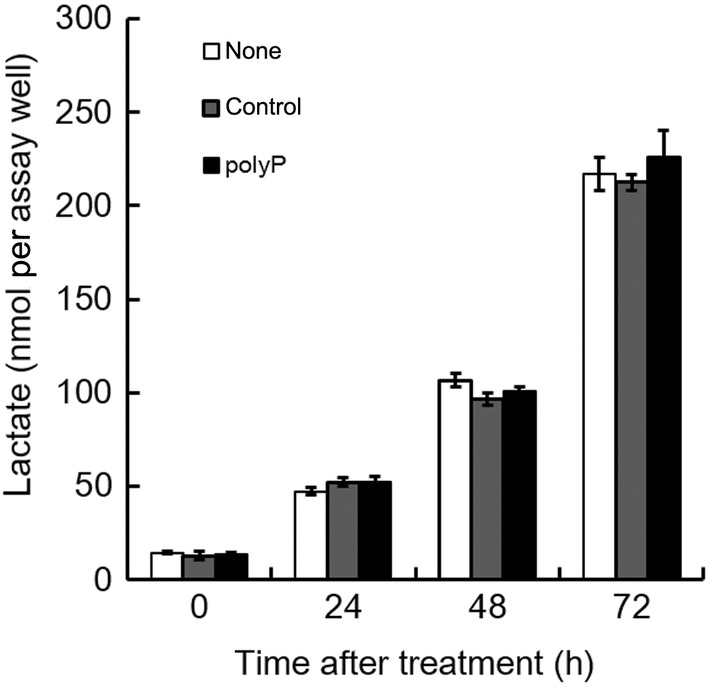
Comparison of lactate secretion in H1299 cells. Lactate secretion following polyP treatment was monitored using the Lactate Colorimetric Assay Kit II (BioVision Inc.). The white bar represents the untreated case (none), the gray bar represents 1 mm sodium phosphate (pH 6.8) as a control for polyP, and the black bar represents 1 mm polyP. Values represent the mean (± SD) of sextuplicate experiments.

In normal cells, polyP has been identified as a regulator of the metabolic switch between OXPHOS and glycolysis [8]. The enzymatic depletion of mitochondrial polyP has also been shown to enhance the activation of the pentose phosphate pathway (PPP) [9]. Moreover, in tumor cells, the increase in glucose uptake and lactate secretion due to elevated aerobic glycolysis, known as the Warburg effect, provides a survival advantage for tumors [45]. Thus, we compared lactate secretion between the control groups and polyP‐treated cells, considering the possibility that changes in the cellular ATP content in polyP‐treated cells might have resulted from alterations in cellular metabolism. However, no significant differences were observed between the groups (Fig. 5). These results may be attributed to several factors related to the specific characteristics of cancer cells and their metabolic adaptations. Lactate, which is primarily produced through the conversion of pyruvate by lactate dehydrogenase, was limited in availability in the absence of pyruvate. Therefore, the effect of polyP on lactate secretion may be attenuated when pyruvate is lacking in the culture medium. However, it is important to note that different cancer cell lines may respond differently to polyP treatment, and various factors in the cellular microenvironment can influence lactate secretion. Pirttiniemi *et al*. [46] found that cells treated with long‐chain polyP (P700) treatment is associated with the activation of mTOR activation in human leukocytes and Wang *et al*. [26] reported that polyP stimulates mTOR in mammary cancer cells. It is well known that mTOR activity plays a central role in energy homeostasis, coordinating protein synthesis, cell growth and proliferation, metabolic intermediate generation, and mitochondrial biogenesis and functions [47]. The depletion of ATP in polyP‐treated cells in the present study might be linked to the mTOR‐related pathway, which can influence the metabolic fate and ATP production, potentially overriding the effects of pyruvate supplementation on ATP recovery in cancer cells. In addition, cancer cells have been reported to proliferate more rapidly in the presence of exogenous pyruvate compared to lactate [48].

**Fig. 6:**
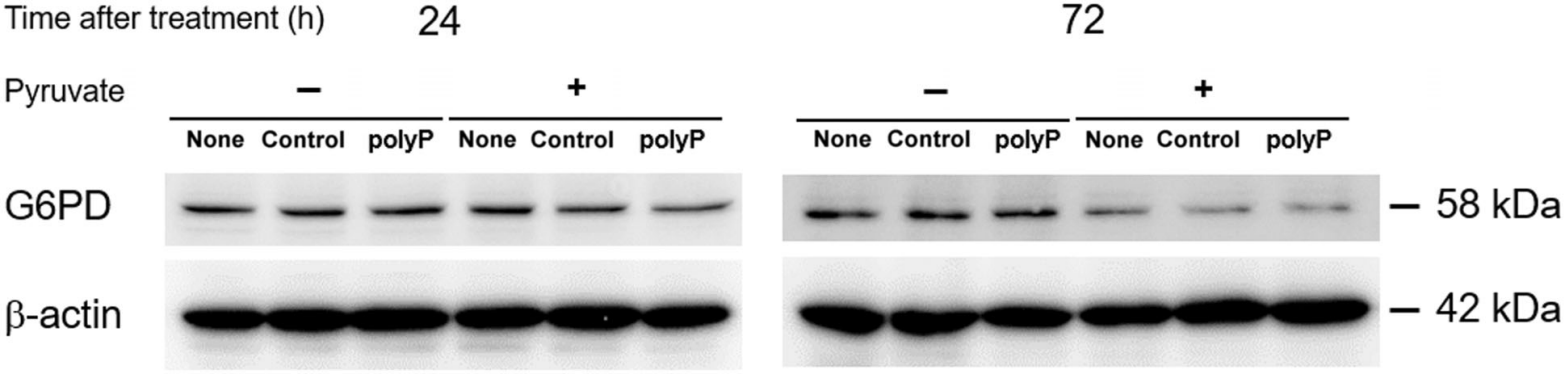
Expression of glucose‐6‐phosphate dehydrogenase (G6PD). The expression levels of G6PD and β‐Actin were assessed by western blotting with or without pyruvate supplementation in the culture medium.

The comparison of the western blotting results revealed that polyP did not affect the activity of glucose‐6‐phosphate dehydrogenase (G6PD) (Fig. 6). Moreover, we observed no discernible shift in metabolism toward the PPP in H1299 cells; however, further experimental validation is required. Hambardikar *et al*. [9] suggested that a lack of polyP in mitochondria enhances the generation of ROS and the activity of PPP through the regulation of nuclear factor erythroid 2‐related factor 2 (Nrf2) in HEK293 cells. While further studies are necessary, the variation observed between HEK293 and H1299 cells in the present study might be attributed to differences in *p53* status or represent different aspects of the same phenomenon. The *p53* tumor suppressor gene has been recognized for its pivotal roles in responding to various cellular stressors, including DNA damage, hypoxia, and oncogenic activation [44]. Recent studies have unveiled its additional roles beyond cellular metabolic alterations through the regulation of energy metabolism and oxidative stress [49, 50]. Thus, variations in the presence or absence of *p53* are expected to lead to distinct energy metabolism and energy supply pathways.

**Fig. 7:**
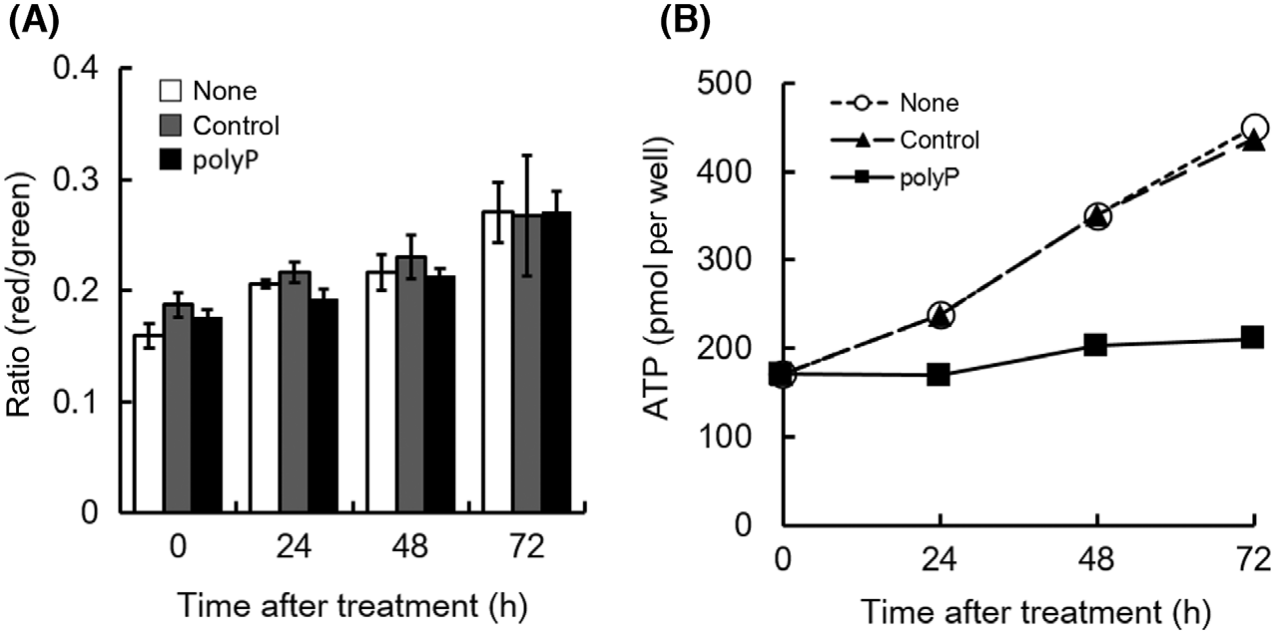
Effect of additive pyruvate on mitochondrial membrane potential (MMP) (A) and cellular ATP content (B). The changes in MMP were compared among three groups: none, control, and polyP. The white bar represents the untreated case (none), the gray bar represents 1 mm sodium phosphate (pH 6.8) as a control for polyP, and the black bar represents 1 mm polyP. The cellular ATP content was measured in H1299 cells with the following representations: white circle (○) for none, black triangle (▲) for 1 mm sodium phosphate, pH 6.8 as a control for polyP, and black square (■) for 1 mm polyP. The values represent the mean (± SD) of sextuplicate experiments.

**Fig. 8:**
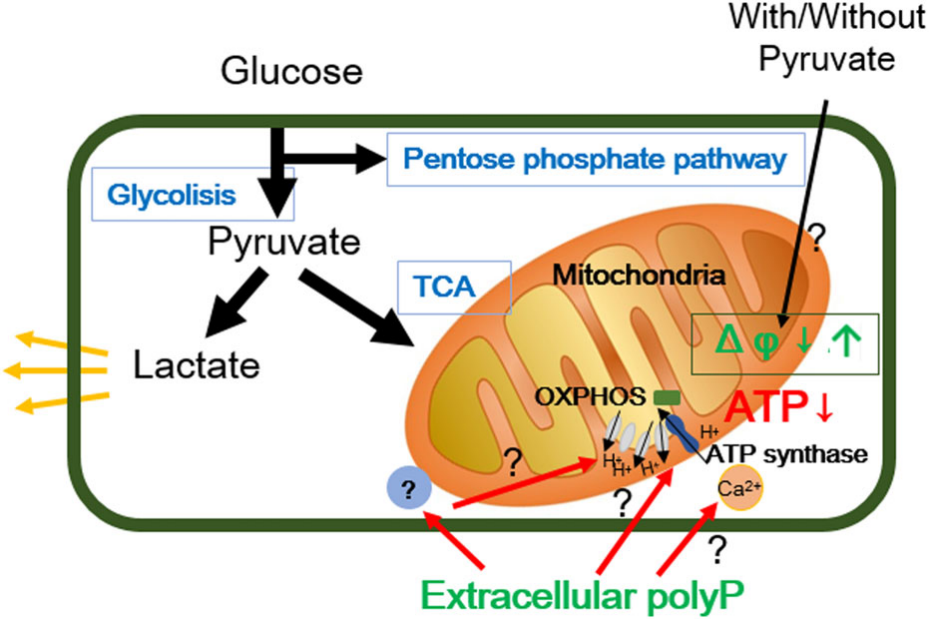
Schematic representation of our hypothesis. PolyP may directly or indirectly regulate the levels of ATP and mitochondrial membrane potential (MMP), thereby influencing the regulation of mitochondrial permeability transition pore (mPTP), enzymatic activity of Fo‐F1 ATP synthase, oxidative phosphorylation (OXPHOS), and cellular Ca^2+^ levels in tumor cells.

Previous studies have shown that various types of cancer cells harbor specific genetic alterations that affect their metabolism and responses to pyruvate. For example, mutations in key enzymes that regulate pyruvate metabolism, such as pyruvate dehydrogenase or lactate dehydrogenase, can affect the conversion of pyruvate to ATP, potentially causing a lack of ATP recovery despite pyruvate supplementation [51]. Interestingly, the addition of pyruvate to the culture medium led to the recovery of the decreased MMP in polyP‐treated cells (Fig. 7A); however, the ATP content in polyP‐treated cells remained depleted, even with the addition of pyruvate (Fig. 7B). In the present study, the cells were cultured in a culture medium without pyruvate. The decrease in MMP observed in polyP‐treated cells can be attributed to the altered metabolic state and energy imbalance resulting from the combined effects of polyP and pyruvate deprivation. ATP is essential for maintaining the proton gradient across the inner mitochondrial membrane [52, 53]. As ATP levels decrease, the proton gradient is disrupted, leading to a decrease in MMP. In addition, pyruvate plays a key role in the tricarboxylic acid (TCA) cycle, and its absence can impair metabolite flux through the cycle, thereby affecting the electron transport chain and ATP synthesis. This disruption of mitochondrial metabolism can affect the integrity and function of the electron transport chain complex, leading to a reduction in MMP. Further studies are needed to evaluate the precise mechanisms underlying the decrease in MMP in polyP‐treated cells in the absence of pyruvate. According to our hypothesis, in tumor cells, the decrease in MMP caused by the addition of extracellular polyP occurs only under pyruvate‐limited conditions, whereas ATP depletion in polyP‐treated cells occurs independently of pyruvate. PolyP may directly affect the ATPase activity and regulate ATP metabolism (Fig. 8). In the present study, we showed that polyP only inhibits the increase in cellular ATP without affecting the Warburg effect or PPP. Further investigation into the details of this mechanism is necessary; however, polyP appears to play a significant role in regulating the cellular ATP content in tumor cells, thus potentially influencing cellular fate by balancing its intra‐ and extracellular concentrations.

## Conclusion

In the present study, we observed ATP depletion in exogenous polyP‐treated tumor cells, concomitant with a decrease in MMP. However, the addition of polyP did not affect the Warburg effect or glucose metabolism. While further investigation is warranted; the complex interplay among polyP, ATP metabolism, OXPHOS, Ca^2+^, MMP, and cellular energy metabolism may offer potential avenues for therapeutic intervention.

## Materials and methods

### Cell culture and reagents

The human non‐small cell lung cancer cell line, H1299, was obtained from the American Type Culture Collection (ATCC, Manassas, VA, USA) and maintained in Dulbecco's modified Eagle's medium (DMEM; Sigma‐Aldrich Co., St. Louis, MO, USA) with high glucose and without pyruvate. The human glioblastoma cell line T98G was obtained from the RIKEN BioResource Research Center (RIKEN BRC, Tokyo, Japan) and maintained in Roswell Park Memorial Institute 1640 medium (RPMI 1640; Sigma‐Aldrich Co.). The culture media for both cell lines were supplemented with 10% fetal bovine serum (FBS; Cansera, ON, Canada), 100 unit·mL^−1^ penicillin, and 100 μg·mL^−1^ streptomycin (Sigma‐Aldrich Co.) and maintained at 37 °C in a humidified atmosphere containing 5% CO_2_. PolyPs, with an average chain length of 120, were generously provided from Dr. Shiba (RegeneTiss Inc, Tokyo, Japan). The culture medium was changed every 3 days. As a control for polyP treatment, the culture medium mentioned above was used with the same volumes of exogenous polyP, Milli‐Q water (none), and sodium phosphate buffer (pH 6.9) (control). The cells treated with Milli‐Q water and sodium phosphate buffer were used as negative control groups. Pyruvate (FUJIFILM Wako Pure Chemical Corp., Osaka, Japan) and 200 nm cyclosporine A (CsA; FUJIFILM Wako Pure Chemical Corp.) were supplemented in the indicated experiments at concentrations of 100 mmol·L^−1^ and 200 nm, respectively.

### Cell growth

Approximately 3000 cells were seeded in each well of a 96‐well plate. After 24 h of incubation, the medium was replaced with the medium supplemented with MilliQ water (none), 1 mm sodium phosphate (pH 6.8, Control), or 1 mm of long chain length of polyP (PolyP). Cell growth was monitored using an MTS assay (CellTiter 96^®^ AQueous One Solution Cell Proliferation Assay; Promega Corporation, Madison, WI, USA). The absorbance was measured at 490 nm using a Model 680 Microplate Reader (Bio‐Rad Laboratories, Inc., Hercules, CA, USA).

### Adenosine triphosphate assay

For the adenosine triphosphate (ATP) assay, approximately 3000 cells were seeded in each well of a 96‐well plate overnight. Subsequently, the cells were treated with either 1 mm sodium phosphate (pH 6.8) or 1 mm polyP for the indicated durations. The change in cellular ATP content in each well was measured using the CellTiter‐Glo^®^ 2.0 Assay kit (Promega Corporation). Chemical luminescence was detected using the AVRO MX instrument (PerkinElmer Inc., Waltham, MA, USA).

### Mitochondrial membrane potential assay

For the MMP assay, approximately 3000 cells were seeded in each well of a 96‐well plate for 24 h. Subsequently, the culture medium was replaced with the fresh medium supplemented with 10% FBS containing 1 mm sodium phosphate (pH 6.8) or 1 mm polyP. At the indicated time points, the Cell Meter™ JC‐10 reagent (AAT Bioquest, Inc., Sunnyvale, CA, USA) was added to the culture medium and incubated for 30 min at 37 °C in 5% CO_2_. Fluorescent intensities at Ex/Em = 490/535 for green and Ex/Em = 490/572 for red were detected using the AVRO MX instrument (PerkinElmer, Inc.). The MMP was estimated as a red/green ratio.

### Lactate assay

For the lactate assay, approximately 3000 cells were seeded into each well of a 96‐well plate for 24 h. Subsequently, the culture medium was replaced with the fresh medium supplemented with 10% FBS and 1 mm sodium phosphate (pH 6.8) or 1 mm polyP. At the indicated time points, the supernatants of the culture medium were centrifuged at 320 ***g*** and collected in the new tube and then immediately frozen in liquid nitrogen and stored at −80 °C. Lactate content was measured by a colorimetric assay at an absorption wavelength of 450 nm using a Lactate Colorimetric Assay Kit II (BioVision Inc., Milpitas, CA, USA).

### Western blotting

The cells were lysed in a lysis buffer containing 50 mm Tris–HCl (pH 7.4), 150 mm NaCl, 5 mm MgCl_2_, 1% NP‐40, 0.1% SDS, 0.5% sodium deoxycholate, 1 mm Na_3_VO_4_, and a complete protease inhibitor cocktail (Roche, Indianapolis, IN, USA) after treatment with polyP for 24 and 72 h. The supernatants were clarified by microcentrifugation. After adjusting the protein concentration, the protein samples were subjected to sodium dodecyl sulfate‐polyacrylamide gel electrophoresis (SDS/PAGE), and the separated proteins were transferred to polyvinylidene difluoride membranes (Bio‐Rad Laboratories). The membranes were incubated with primary antibodies specific for G6PD (1 : 1000 dilution; Cell Signaling Technology, Inc., Danvers, MA, USA) and β–actin (1 : 5000 dilution; Sigma‐Aldrich, Inc.), followed by peroxidase‐labeled secondary antibodies. Signals were developed using ECL Western Blotting Detection Reagent (GE Healthcare, Little Chalfont, UK), and the luminescent signals were detected using the ChemiDoc™ MP Imaging System (Bio‐Rad Laboratories).

### Statistical analysis

All statistical analyses were performed using spss statistics version 18 (IBM Corp., Armonk, NY, USA). Comparisons between the three groups were performed using the multiple comparison procedure with Tukey's honestly significant difference test. The error bars represent the standard deviation (SD) values. Statistical significance was set at *P* < 0.05.

## Conflict of interest

The authors declare no conflict of interest.

### Peer review

The peer review history for this article is available at https://www.webofscience.com/api/gateway/wos/peer‐review/10.1002/2211‐5463.13753.

## Author contributions

Conceptualization, KT; data curation, KT, TT, MH, NM, and YG; formal analysis, KT and MH; funding acquisition, KT; investigation, KT, TT, MH, NM, and YG; methodology, KT; project administration, KT; resources, KT; software, KT, MH, NM, and YG; supervision, KT; validation, KT; visualization, KT, MH, NM, and YG; writing—original draft preparation, KT and TT; writing—review and editing, KT. All authors have read and agreed to the published version of the manuscript.

## Data Availability

The data that support the findings of this study are available from the corresponding author [tsutsumi@hs.hokudai.ac.jp] upon reasonable request.
